# Supplementary material for: ATRA influences the differentiation and fusion of myoblasts by regulating Rarα/Pitx2, leading to abnormal development of the pelvic floor muscles (PFMs) in fetal rats
Source: PLoS One. 2026 Apr 17;21(4):e0345764. doi: 10.1371/journal.pone.0345764 (PMC13089754; doi:10.1371/journal.pone.0345764)
Supplement: S1 Fig — (a) L6 cells were induced to differentiate for various lengths of time. Western blotting analysis showing Rarαand Pitx2 expression at various stages. D0 indicates undifferentiated L6 cells, and D1 to D4 indicate L6 cells on Days 1–4, respectively, after initial exposure to differentiation medium. (b, c) Quantification of Rarαand Pitx2 protein expression in (a); (d, e) Differentiated medium (CON), vacuum virus, Rarα inhibitory adenovirus, and AM580 effect on Rarα; *p < 0.05, ***p < 0.001, ****p < 0.0001. (DOCX) [file pone.0345764.s001.docx]

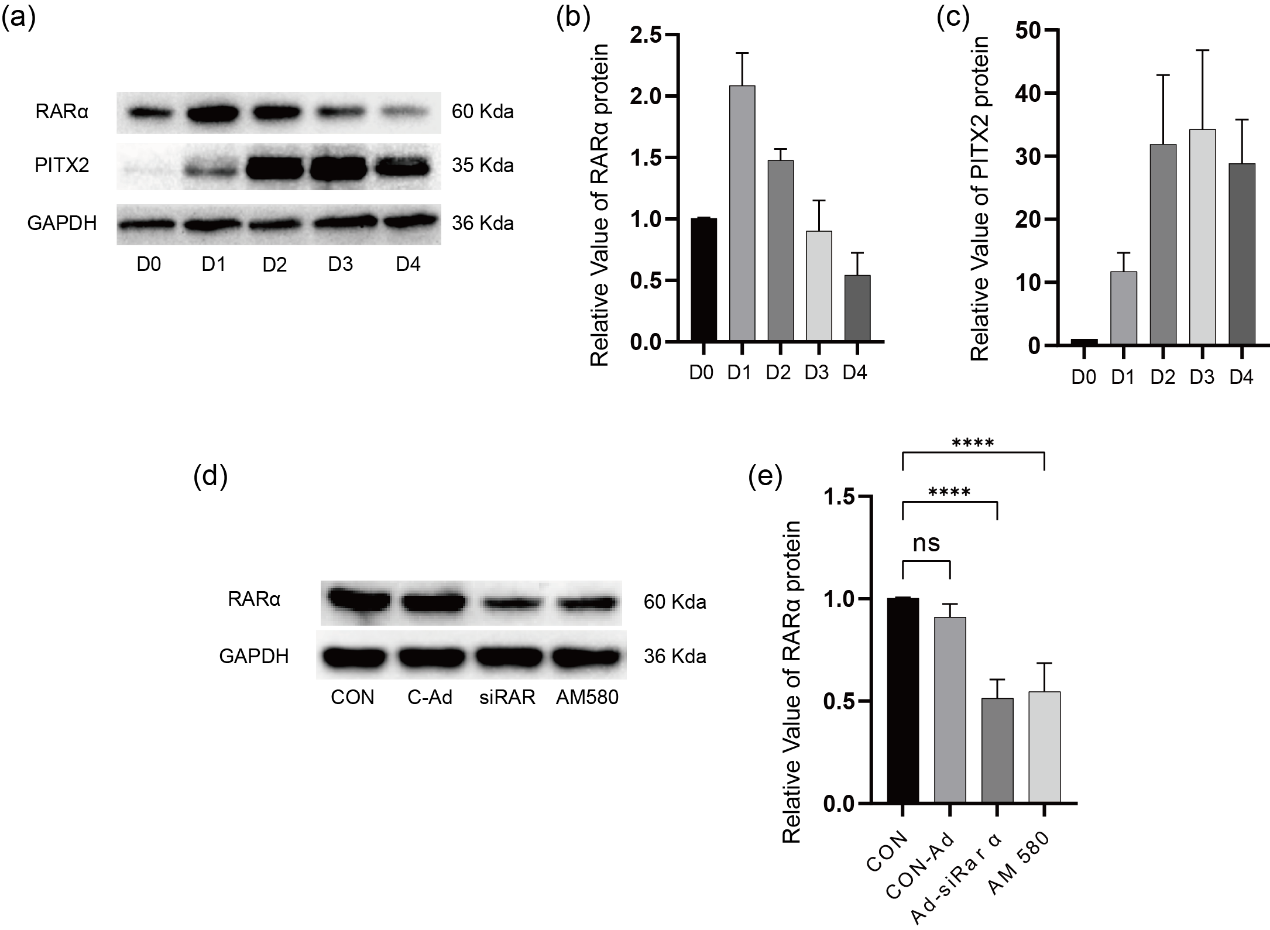


**Fig1 Effects of various treatments on Rarα in L6 cells.**

(a) L6 cells were induced to differentiate for various lengths of time. Western blotting analysis showing Rarαand Pitx2 expression at various stages. D0 indicates undifferentiated L6 cells, and D1 to D4 indicate L6 cells on Days 1–4, respectively, after initial exposure to differentiation medium. (b, c) Quantification of Rarαand Pitx2 protein expression in (a); (d, e) Differentiated medium (CON), vacuum virus, Rarα inhibitory adenovirus, and AM580 effect on Rarα; *p＜0.05, ***p＜0.001, ****p＜0.0001.
