# Supplementary material for: ATRA influences the differentiation and fusion of myoblasts by regulating Rarα/Pitx2, leading to abnormal development of the pelvic floor muscles (PFMs) in fetal rats
Source: PLoS One. 2026 Apr 17;21(4):e0345764. doi: 10.1371/journal.pone.0345764 (PMC13089754; doi:10.1371/journal.pone.0345764)
Supplement: S2 Fig — (a, b) Among the three plasmids, sh2 exhibited the most significant inhibitory effect on Pitx2. (c, d) Differentiated medium (CON), vacuum virus, Pitx2 overexpression adenovirus, and blank plasmid, Pitx2 inhibitory plasmid effect on Pitx2. *p < 0.05, ***p < 0.001, ****p < 0.0001. (DOCX) [file pone.0345764.s002.docx]

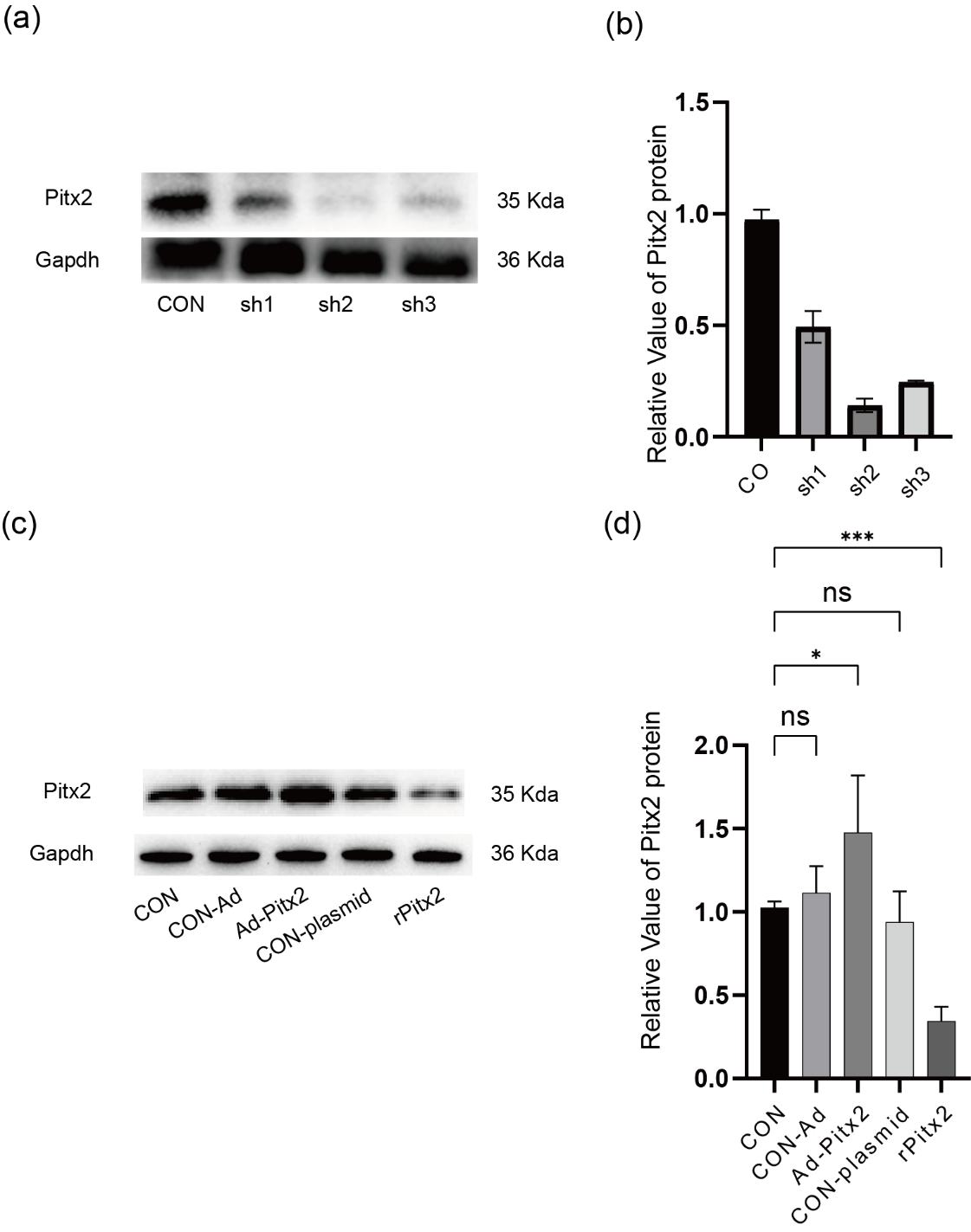


**Fig2 The effect of different plasmids on Pitx2 expression in L6 cells.**

(a, b) Among the three plasmids, sh2 exhibited the most significant inhibitory effect on Pitx2. (c, d) Differentiated medium (CON), vacuum virus, Pitx2 overexpression adenovirus, and blank plasmid, Pitx2 inhibitory plasmid effect on Pitx2. *p＜0.05, ***p＜0.001, ****p＜0.0001.
