# Supplementary material for: ATRA influences the differentiation and fusion of myoblasts by regulating Rarα/Pitx2, leading to abnormal development of the pelvic floor muscles (PFMs) in fetal rats
Source: PLoS One. 2026 Apr 17;21(4):e0345764. doi: 10.1371/journal.pone.0345764 (PMC13089754; doi:10.1371/journal.pone.0345764)
Supplement: S1 Table — (DOCX) [file pone.0345764.s003.docx]

**Table1 Comparison of the number of malformed fetal rats between the control group and the model group**

| Time | E16.5 | E17.5 | E18.5 | E19.5 | E20.5 | sum |
| --- | --- | --- | --- | --- | --- | --- |
| Control group  (malformation/sum) | 0/45 | 0/44 | 0/48 | 0/50 | 0/48 | 378  (0/378) |
| Model group  (malformation/sum) | 46/46 | 46/46 | 49/49 | 41/41 | 36/38 | 361  (359/361) |
