# Supplementary material for: ATRA influences the differentiation and fusion of myoblasts by regulating Rarα/Pitx2, leading to abnormal development of the pelvic floor muscles (PFMs) in fetal rats
Source: PLoS One. 2026 Apr 17;21(4):e0345764. doi: 10.1371/journal.pone.0345764 (PMC13089754; doi:10.1371/journal.pone.0345764)
Supplement: S3 Fig — (a) Sagittal section of E17.5 fetal rats in the normal group. (b) Sagittal section of ARM group. (c, d, e) Horizontal sections of fetal rats at E16.5, E17.5, and E18.5 in the normal group. (f, g, h) Horizontal sections of fetal rats at E16.5, E17.5, and E18.5 in the ARM group. (DOCX) [file pone.0345764.s004.docx]

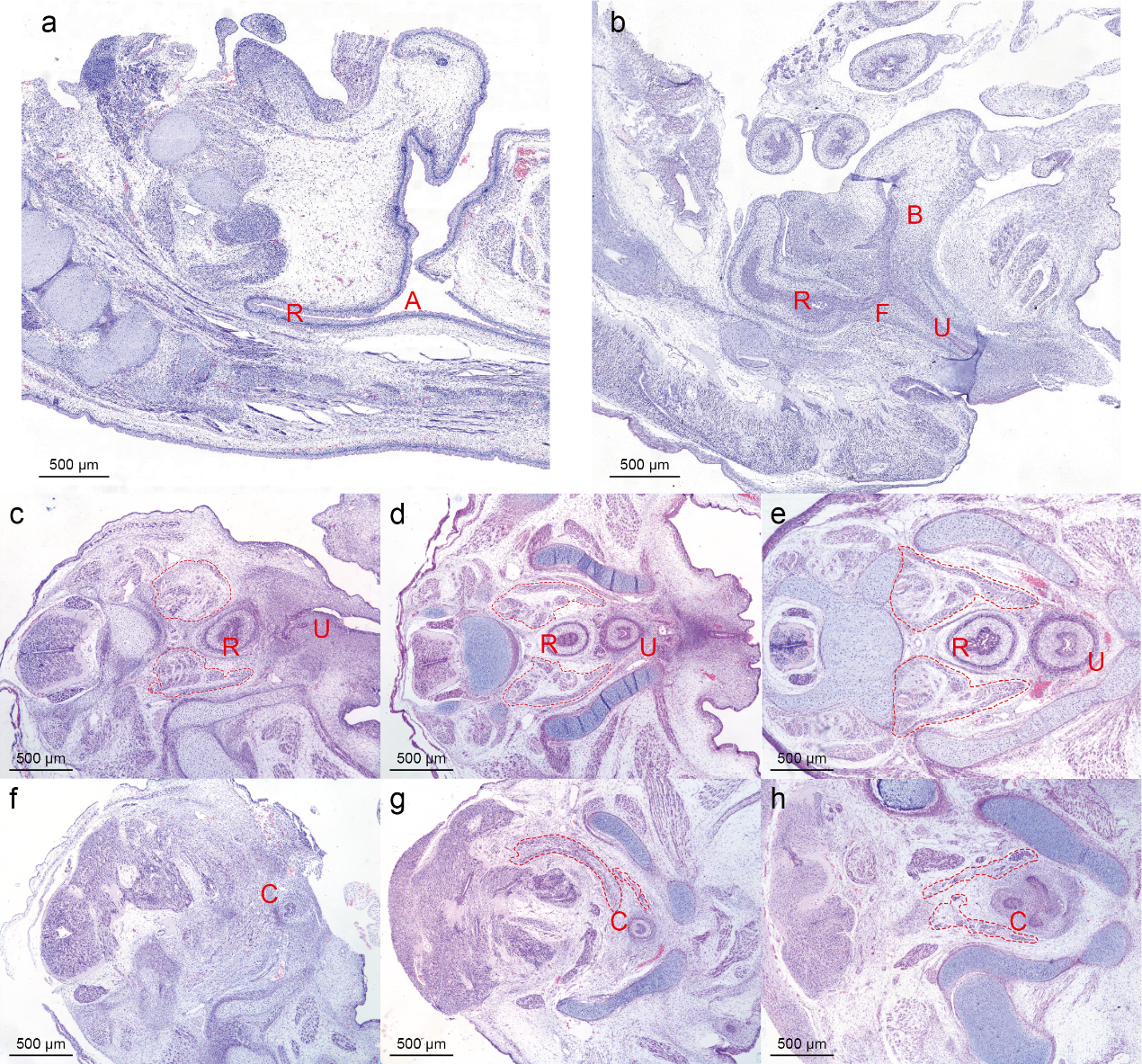


**Fig 3 HE staining of fetal rat sections (magnification, 40×), R: rectum, A: anus, F: fistula, B: bladder, U: urethra, C: cloaca, Red dashed lines indicate muscle areas.**

(a) Sagittal section of E17.5 fetal rats in the normal group. (b) Sagittal section of ARM group. (c, d, e) Horizontal sections of fetal rats at E16.5, E17.5, and E18.5 in the normal group. (f, g, h) Horizontal sections of fetal rats at E16.5, E17.5, and E18.5 in the ARM group.
