# Supplementary figures and images for: ATRA influences the differentiation and fusion of myoblasts by regulating Rarα/Pitx2, leading to abnormal development of the pelvic floor muscles (PFMs) in fetal rats
Source: PLoS One. 2026 Apr 17;21(4):e0345764. doi: 10.1371/journal.pone.0345764 (PMC13089754; doi:10.1371/journal.pone.0345764)

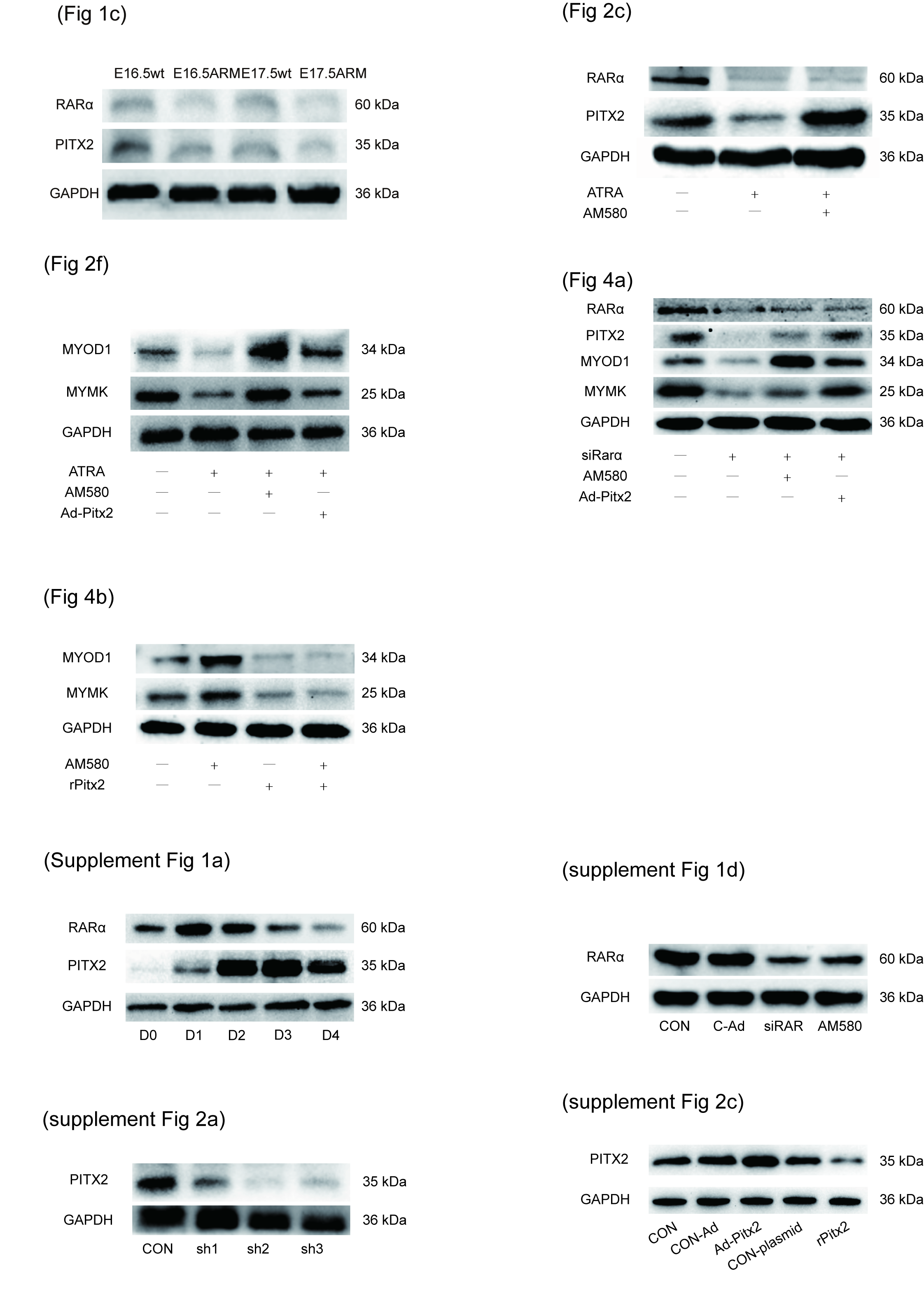

Supplement: S1 File — (TIF) [file pone.0345764.s005.tif]
