# Supplementary material for: ATRA influences the differentiation and fusion of myoblasts by regulating Rarα/Pitx2, leading to abnormal development of the pelvic floor muscles (PFMs) in fetal rats
Source: PLoS One. 2026 Apr 17;21(4):e0345764. doi: 10.1371/journal.pone.0345764 (PMC13089754; doi:10.1371/journal.pone.0345764)
Supplement: Attachment — Submitted filename: Response to Reviewer.docx [file pone.0345764.s007.docx]

Dear Editor of PLOS one:

We sincerely thank the editor and all reviewers for their valuable feedback that we have used to improve the quality of our manuscript. The reviewer comments are laid out below in italicized font and specific concerns have been numbered. Our response is given in normal font and changes/additions to the manuscript are given in the red text.

Journal requirements:

When submitting your revision, we need you to address these additional requirements.

*1. Please ensure that your manuscript meets PLOS ONE's style requirements, including those for file naming. The PLOS ONE style templates can be found at*

*https://journals.plos.org/plosone/s/file?id=wjVg/PLOSOne_formatting_sample_main_body.pdf and*

*https://journals.plos.org/plosone/s/file?id=ba62/PLOSOne_formatting_sample_title_authors_affiliations.pdf*

**Answer1:** Changes have been made as required.

*2. To comply with PLOS ONE submissions requirements, in your Methods section, please provide additional information regarding the experiments involving animals and ensure you have included details on (1) methods of sacrifice, (2) methods of anesthesia and/or analgesia, and (3) efforts to alleviate suffering.*

**Answer2:** The corresponding section has been added to the manuscript.

*3. Thank you for stating the following financial disclosure:*

*“This study was supported by the Program for Youth Innovation in Future Medicine, Chongqing Medical University (grant No. W0125).”*

*Please state what role the funders took in the study.  If the funders had no role, please state: "The funders had no role in study design, data collection and analysis, decision to publish, or preparation of the manuscript."*

*If this statement is not correct you must amend it as needed.*

*Please include this amended Role of Funder statement in your cover letter; we will change the online submission form on your behalf.*

**Answer3:** The corresponding section has been added to the manuscript.

*4. PLOS ONE now requires that authors provide the original uncropped and unadjusted images underlying all blot or gel results reported in a submission’s figures or Supporting Information files. This policy and the journal’s other requirements for blot/gel reporting and figure preparation are described in detail at https://journals.plos.org/plosone/s/figures#loc-blot-and-gel-reporting-requirements and https://journals.plos.org/plosone/s/figures#loc-preparing-figures-from-image-files. When you submit your revised manuscript, please ensure that your figures adhere fully to these guidelines and provide the original underlying images for all blot or gel data reported in your submission. See the following link for instructions on providing the original image data: https://journals.plos.org/plosone/s/figures#loc-original-images-for-blots-and-gels.*

*In your cover letter, please note whether your blot/gel image data are in Supporting Information or posted at a public data repository, provide the repository URL if relevant, and provide specific details as to which raw blot/gel images, if any, are not available. Email us at plosone@plos.org if you have any questions.*

**Answer4:** All the original files of the blots are included in the "supplement" file.

*5. We note that you have indicated that there are restrictions to data sharing for this study. For studies involving human research participant data or other sensitive data, we encourage authors to share de-identified or anonymized data. However, when data cannot be publicly shared for ethical reasons, we allow authors to make their data sets available upon request. For information on unacceptable data access restrictions, please see http://journals.plos.org/plosone/s/data-availability#loc-unacceptable-data-access-restrictions.*

*Before we proceed with your manuscript, please address the following prompts:*

*a) If there are ethical or legal restrictions on sharing a de-identified data set, please explain them in detail (e.g., data contain potentially identifying or sensitive patient information, data are owned by a third-party organization, etc.) and who has imposed them (e.g., a Research Ethics Committee or Institutional Review Board, etc.). Please also provide contact information for a data access committee, ethics committee, or other institutional body to which data requests may be sent.*

*b) If there are no restrictions, please upload the minimal anonymized data set necessary to replicate your study findings to a stable, public repository and provide us with the relevant URLs, DOIs, or accession numbers. Please see http://www.bmj.com/content/340/bmj.c181.long for guidelines on how to de-identify and prepare clinical data for publication. For a list of recommended repositories, please see https://journals.plos.org/plosone/s/recommended-repositories. You also have the option of uploading the data as Supporting Information files, but we would recommend depositing data directly to a data repository if possible.*

*Please update your Data Availability statement in the submission form accordingly.*

**Answer5:** All the original files are included in the "supplement" file.

*6. Your ethics statement should only appear in the Methods section of your manuscript. If your ethics statement is written in any section besides the Methods, please delete it from any other section.*

**Answer6:** Changes have been made as required.

*7. Please include a separate caption for each figure in your manuscript.*

**Answer7:** Changes have been made as required.

*8. We are unable to open your Supporting Information file [supplement.zip]. Please kindly revise as necessary and re-upload.*

**Answer8:** The file has been re-uploaded.

*9. Please include captions for your Supporting Information files at the end of your manuscript, and update any in-text citations to match accordingly. Please see our Supporting Information guidelines for more information: http://journals.plos.org/plosone/s/supporting-information.*

**Answer9:** Changes have been made as required.

Reviewer #1:

*Major points:
1/ Figure 2 - for experiments based on L6 cells, the authors use ATRA at 10µM and Am580 at 20nM. Both ligands are high-affinity agonists for RARa, with Am580 having the best Kd (approx. 1 Log difference). Such concentrations pose a problem for the interpretation of results. Given the respective affinities of ATRA and Am580 for RARa, it is likely that when the two molecules are combined for cell treatment, ATRA is bound to RARa and, by competition, prevents binding of Am580. In this case, the observed effect of Am580 could not be attributed to RARa activation. However, despite the competition between ATRA and Am580 to bind RARa, if a fraction of RARa is bound to and activated by Am580, why would ATRA used alone not cause the same effects as Am580, since ATRA is a RARa agonist like Am580?
Taken together, these considerations make the mechanistic interpretation of the results presented in figure 2 difficult. Without calling the results into question, the mechanisms proposed by the authors should be reassessed.*

**Answer1:** In our experiment, we initially used high concentrations of ATRA to stimulate L6 cells, followed by a switch to differentiation medium containing AM580 to further stimulate the cells. Previous studies have shown that the biological effects of ATRA are highly concentration-dependent. Excessive exposure to ATRA can not only reduce RARα expression levels but also promote ubiquitination and degradation of the RARα protein by activating the proteasome pathway, thereby exerting negative feedback inhibition on the signaling pathway. Therefore, after high concentrations of ATRA caused a decrease in RARα levels in L6 cells, subsequent stimulation with AM580 enhanced the effects of ATRA on these cells by acting on the remaining RARα.

*Minor points:
2/ Did the authors test ATRA at concentrations below 10µM in the assays presented? Defining whether a lower concentration of ATRA is capable of mediating comparable effects could be informative about ATRA's mechanism of action. Also it should be noted that a concentration of 10µM, ATRA can bind to other cellular targets (PPARs, RORs, others) and regulate pathways controlled by these proteins.*

**Answer2:** We tested lower concentrations of ATRA (<10 nM) and found that it can increase RARα expression. This study was based on human specimens from previous research. Earlier experiments did not detect changes in RARγ and RARβ expression in the terminal rectum of children with ARM. Therefore, this study focused solely on RARα expression. This represents a limitation of the current study, and we plan to conduct more comprehensive experiments in the future.

*3/ A contradiction is observed between Fig2c and Fig2e. The western blot shows an increase in Pitx2 levels (Fig2c) for ATRA-Am580 co-treatment compared with untreated, whereas quantification of this western blot indicates a decrease.*

**Answer3:** The result shown in Figure 2e is based on the ratio of the target protein expression level to the GAPDH expression level depicted in Figure 2c. Visually, the concentration of the Pitx2 band in the untreated group in Figure 2c appears low; however, the GAPDH level in the same sample is also low, which explains this outcome.

*4/ Why is Am580 not used on its own (Fig. 2)? This experimental condition would constitute a relevant control.*

**Answer4:** As described in Answer 1, AM580 stimulation was applied to L6 cells only after ATRA stimulation; therefore, we did not include results for L6 cells stimulated with AM580 alone.

*5/ Another point is that in vitro a fraction of ATRA can be converted to 9cRA, an agonist for RARs but also for RXR, the heterodimerization partner of RARs. Simultaneous activation of both RAR and RXR partners by an agonist results in transcriptional over-activation, which can bias assays. On the other hand, as RXR is also the heterodimerization partner of other nuclear receptors, its activation by an agonist like 9CRA is likely to affect these other signaling pathways. It would be advisable to check whether 10µM of the ATRA solution used for these studies does not exhibit RXR agonist activity.*

**Answer5:** We will continue the experiment to detect the expression level of RXR in the terminal rectum of children with ARM and to investigate the effect of ATRA on RXR expression in L6 cells.

*6/ Am580 is considered a selective agonist for RARa over RARb and RARg. However, to respect this specificity, it is necessary to be cautious about the amount of ligand used. It is necessary not to exceed a concentration of 10nM of Am580. 20nM is a little too strong. For experiments using cells in culture, 5nM Am580 is sufficient to fully activate RARa.*

**Answer6:** The concentrations of ATRA and AM580 used in this study were evaluated using the CCK-8 assay to determine their effects on L6 cells, and the IC50 values were calculated. Subsequently, we will test the effects of varying concentrations of AM580 on L6 cells.

*7/ In the discussion section, authors mention the response elements (RARE) that enable RARs to associate with DNA in regions regulating target gene expression. They write “RarE sequences”. This term and abbreviation should be defined for readers unfamiliar with this field.*

**Answer7:** Already noted in the article.

*8/ Authors write “chemically synthesized shRNA”. What does the term “chemically” refer to in this context?*

**Answer8:** The shRNA was designed and synthesized by a third-party company; therefore, the related description has been removed from the article.

*9/ Authors use trivial name for RARalpha as well for other nuclear receptors. A logical numbering system and receptor code, supporting the trivial names, was made by the International Committee of Pharmacology Committee on Receptor Nomenclature and Classification (NC-IUPHAR). In each manuscript dealing with nuclear receptors, it is recommended that the receptors be identified by the official names at least once in the summary and the introduction. Once the name has been established, authors may use the trivial name for the remainder of the manuscript. For instance, the trivial name and the formal nomenclature for RARalpha is NR1B1.*

**Answer9:** The corresponding content has been added to the abstract.

*Reviewer #2:*

*The most exciting experiment remains to be done. Having shown the strong effects of siRara on MyoD levels, and the ability of Pitx2 to restore MyoD and Mymk, analyzing the differentiation of L6 myoblasts in these contexts (as in Fig. 3b and c) would provide strong support for the authors' conclusions and strengthen the manuscript.*

**Answer1:** Because the only non-fluorescent plasmid or adenovirus we customized is Ad-Pitx2, we only performed immunofluorescence experiments on cells treated with ATRA+Ad-Pitx2.

I would like to take this opportunity to once again express my sincere gratitude to the editors and reviewers, and I extend my best wishes for your continued happiness and well-being!

                                                  Sincerely Yours,
                                                  Dr. Yi Wang
